# Supplementary material for: Self-organization of human dorsal-ventral forebrain structures by light induced SHH
Source: Nat Commun. 2021 Nov 19;12:6768. doi: 10.1038/s41467-021-26881-w (PMC8604999; doi:10.1038/s41467-021-26881-w)
Supplement: Supplementary file 1 — Supplementary Information [file 41467_2021_26881_MOESM1_ESM.pdf]

Self-organization of human dorsal-ventral forebrain structures by light induced SHH

Riccardo De Santis, Fred Etoc, Edwin A. Rosado-Olivieri, and Ali H. Brivanlou.

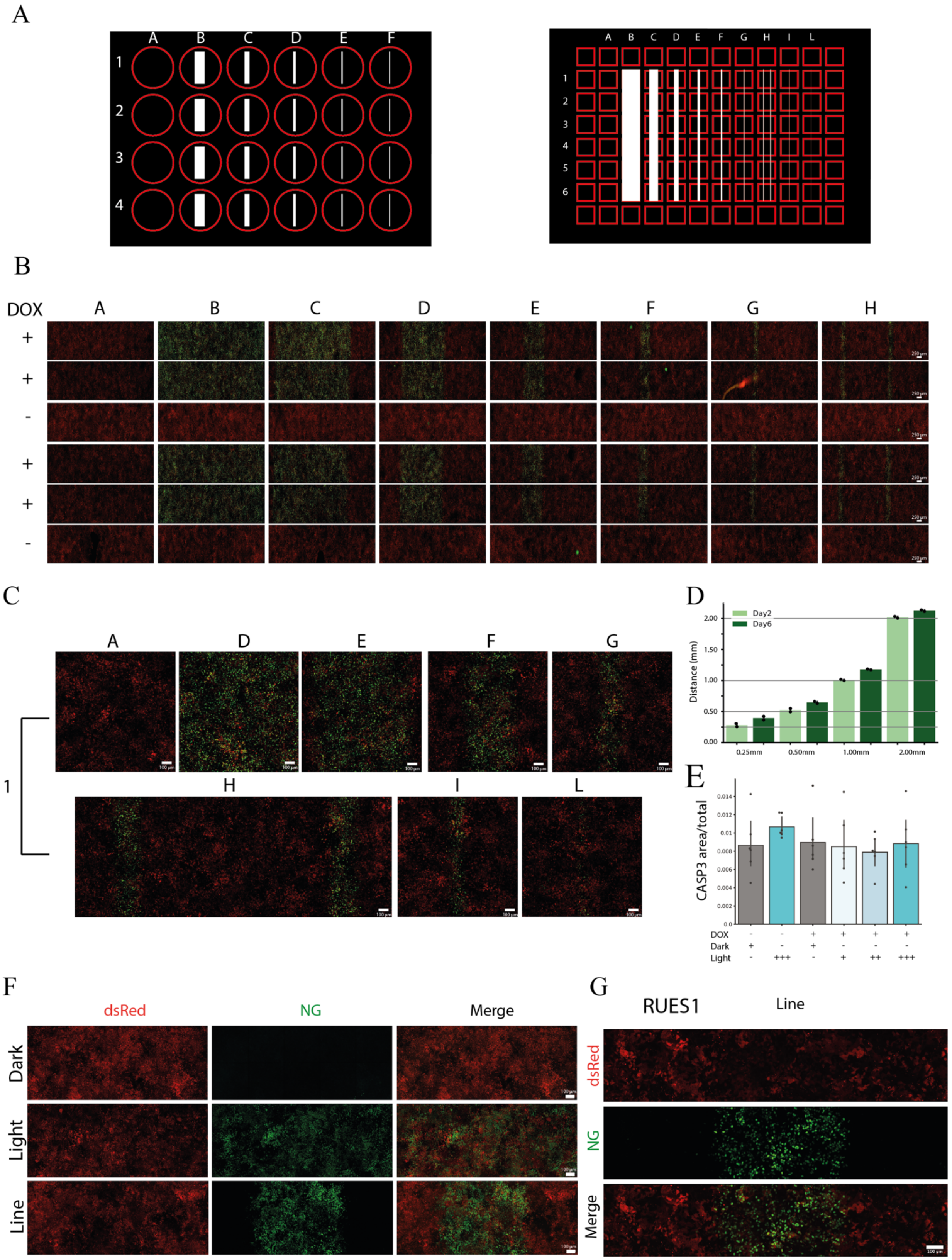
A
A B C D E F
1
2
3
4
A B C D E F G H I L
1
2
3
4
5
6
B
DOX
A B C D E F G H
+
+
-
+
+
-
C
A D E F G
1
H I L
D
Day2
Day6
Distance (mm)
2.00
1.50
1.00
0.50
0.00
0.25mm
0.50mm
1.00mm
2.00mm
E
CASP3 area/total
0.014
0.012
0.01
0.008
0.006
0.004
0.002
0.0
DOX - - + + + +
Dark + - + - - -
Light - +++ - + ++ +++
F
dsRed
NG
Merge
Dark
Light
Line
G
RUES1
Line
dsRed
NG
Merge

Suppl. Fig 1: Systematic and robust light-induced gene expression in hESCs

(A) Blueprint of the film photomask for 24 or 96 multiwells plates with light-permeant aperture. Light permissive regions for 24 well plate: A=0mm, B=4mm, C=2mm, D=1mm, E=0.5mm, F=0.25mm. Light permissive regions for 96 well plate: A=0mm, B=7mm, C=3.5mm, D=2mm, E=1mm, F=0.5mm, G=0.25mm, H=2x 0.25mm, I=0.125mm, L=0.625mm. **(B)** hESC treated with or without DOX and Blue-light for 24h hours, individual wells of a 96 well plate are displayed using the merge of Green and Red. Columns A to H and row 1 to 6, with light aperture: A=0mm, B=7mm, C=3.5mm, D=2mm, E=1mm, F=0.5mm, G=0.25mm, H= 2 lines of 0.25mm. Scale Bar = 250 µm. **(C)** Confocal fluorescence images, Green and Red show the use of light responsive hESC with a 96-well plate set up for light patterning. Selected wells in columns A, D, E, F, G, H, I and L, from row number 1 display photomask patterning after DOX induction and 24h of Blue light. Scale Bar = 100 µm. **(D)** Analysis of the Green module induced territory at different time points (day 2 and day 6) in relation with their corresponding photomask, data are displayed as mean and individual points (n=2 biologically independent samples). **(E)** Quantification of the CASP3 positive area. Conditions: presence or absence of Dox with concomitant Dark or Light stimulation. We titrated the time of light-stimulation using different pulsed light conditions. 1 cycle of pulsed light is equal to 20 seconds of Light-ON and 120 seconds of Light-OFF. The light stimulation intervals are: 1 cycle, 25 cycles, and 600 cycles, data are displayed as mean, SD and individual points (n=5 biologically independent samples). **(F)** Confocal fluorescence images, Green module and Red Module of a 1mm Line induced area at day 6, under full Dark or Light. Red module (dsRed), Green module (NG) and merge composite channel. Scale Bar = 100 µm. **(G)** Validation of optogenetic ePiggyBac vectors to conditionally express a photo-activatable Cre-loxP recombinase in an independent hESCs line, RUES1. Confocal fluorescence images of induced light patterns showing the expression of the Red module (dsRed), Green module (NG) and merge composite channels. Scale Bar = 100 µm.

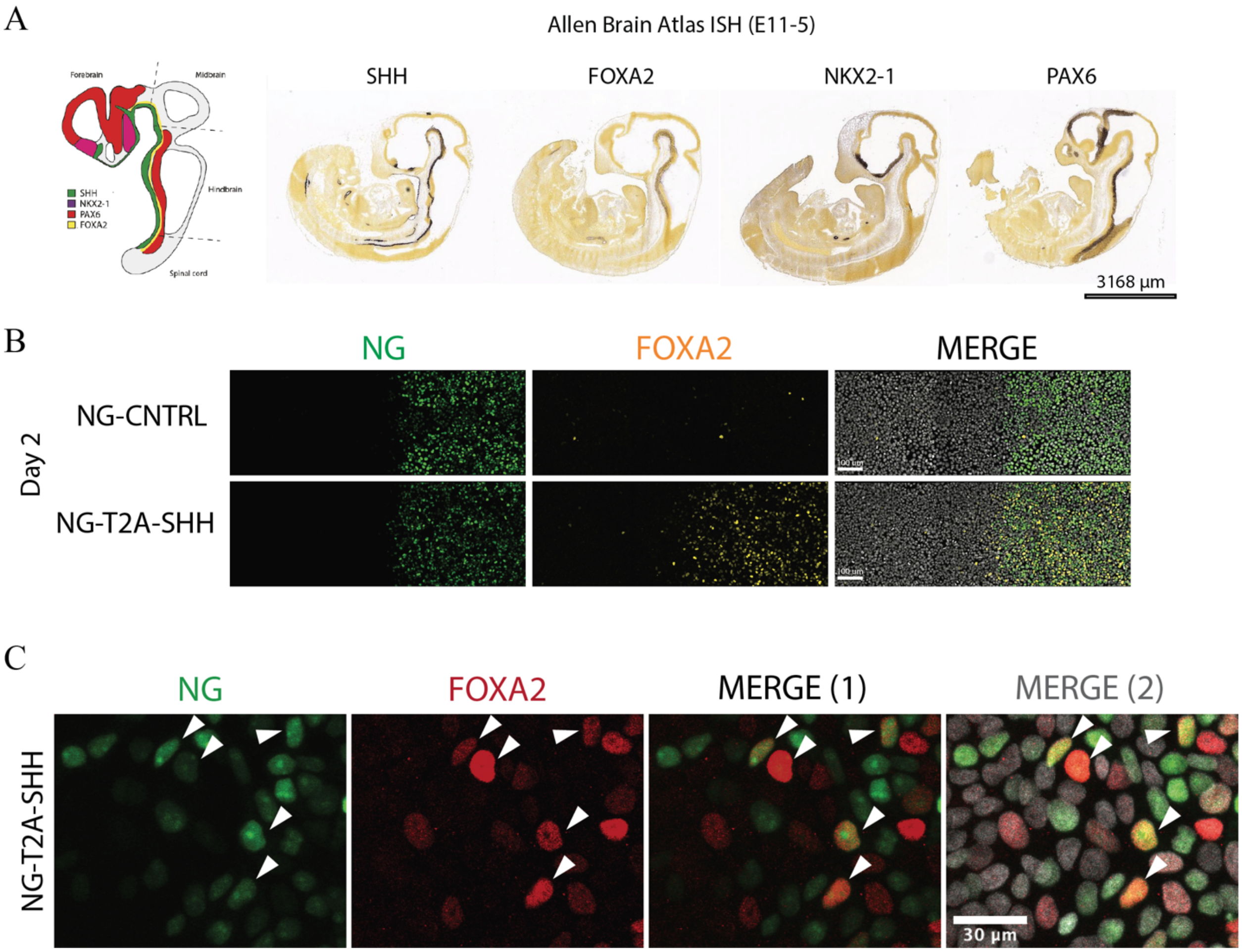

Suppl. Fig 2: Ventral markers and autocrine induction of FOXA2 positive ventral cells upon light-induced SHH

(A) Schematization of mouse embryos in a parasagittal section for Dorso-Ventral (D-V) marker genes (Left panel). Image adapted from Allen brain atlas ISH showing D-V marker genes in the mouse embryo E11.5, Image credit: Allen Institute. (https://developingmouse.brain-map.org) (NKX2-1, SHH, PAX6, FOXA2). Scale bar= 3168 µm. **(B)** Fluorescence images of large fields of view showing consistent FOXA2 induction along the light-induced line of SHH, but not the control NG-CNTRL at day 2. Color code is: NG-Green (Organizer), FOXA2-Yellow, DAPI-Grey and Merge-Composite. Scale bar: 100µm. **(C)** Immunostaining at higher magnification showing double positive NG-SHH and FOXA2 cells. DAPI-Grey, NG-Green, FOXA2-Red and Merge-Composite. Scale bar= 30µm.

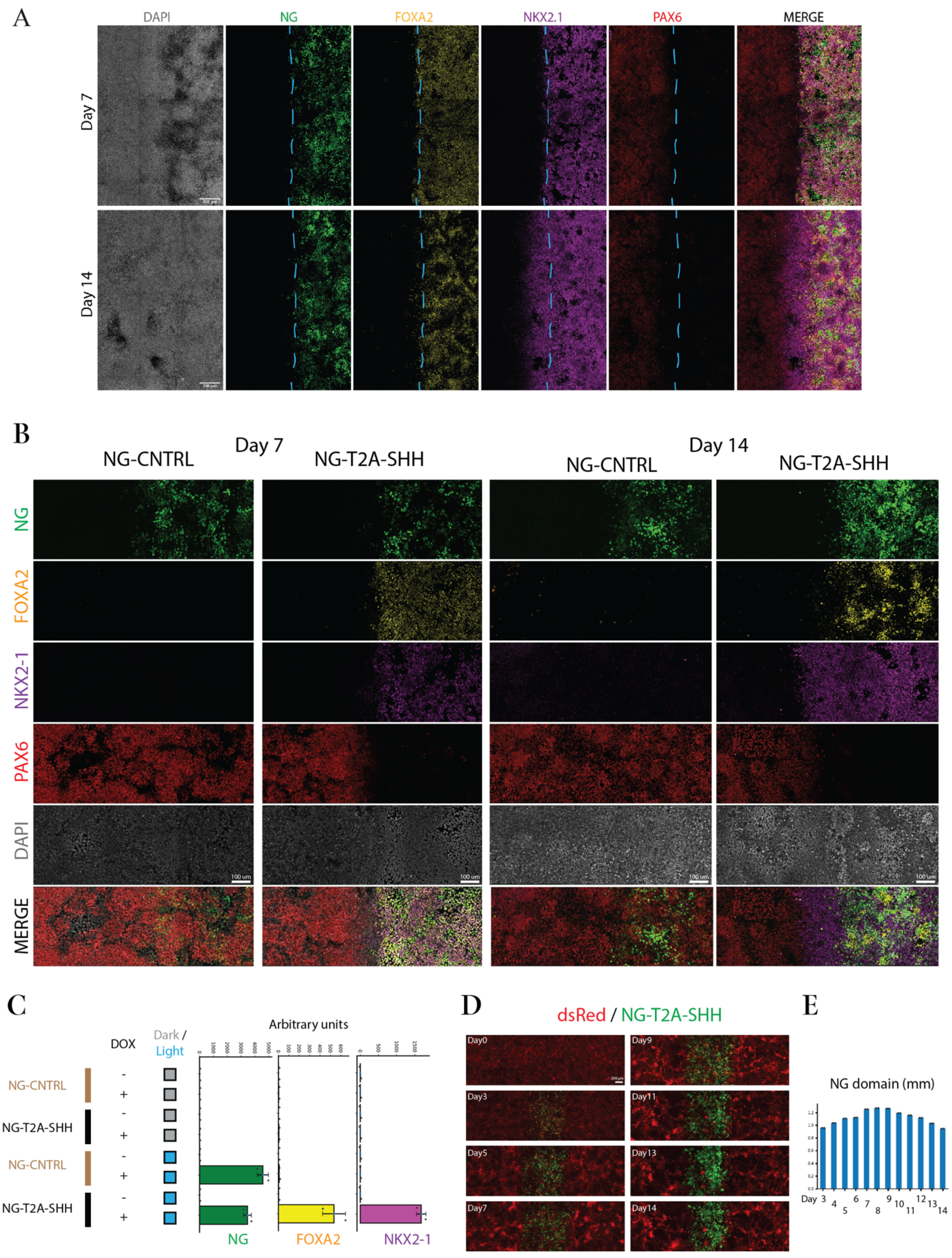

A
DAPI
NG
FOXA2
NKX2.1
PAX6
MERGE
Day 7
Day 14
B
Day 7
Day 14
NG-CNTRL
NG-T2A-SHH
NG
FOXA2
NKX2-1
PAX6
DAPI
MERGE
100 um
C
Arbitrary units
DOX
Dark /
Light
NG-CNTRL
NG-T2A-SHH
NG
FOXA2
NKX2-1
D
dsRed / NG-T2A-SHH
Day0
Day3
Day5
Day7
Day9
Day11
Day13
Day14
E
NG domain (mm)
Day 3 4 5 6 7 8 9 10 11 12 13 14

Suppl. Fig 3: SHH-dependent dorsal-ventral self-organization

(A) Immunofluorescence images of large fields of cells showing consistent patterning along the light-induced line of SHH at day 7 and day 14. Color code is: NG-Green (Organizer), FOXA2-Yellow (Ventral), NKX2-1-Magenta (Ventral), PAX6-Red (Dorsal), Merge-Composite. Dashed cyan line indicate the border of SHH producing cells. Scale bar: 200µm. **(B)** Immunostaining for dorsal-ventral markers in light induced SHH cells and NG-CNTRL during differentiation at different time points, day 7 and 14. DAPI-Grey, NG-Green, FOXA2-Yellow, NKX2-1- Magenta, PAX6-Red and Merge-Composite. Scale bar: 100µm. **(C)** qRT-PCR showing the mRNA level of ventral marker genes (FOXA2 and NKX2-1) specifically induced in the NG-T2A-SHH line but not in the control line, NG-CNTRL. Dark condition and DOX-minus controls do not induce ventral marker genes during differentiation. Histograms display the average, the SD and individual points (n=2 biologically independent samples). D) Time-laps imaging of NG-T2A-SHH cells induced with a 1mm photomask. Images display day: 0,3,5,7,9,11,13,14. dsRed-Red and NG-green. Scale bar 200 µm. E) Analysis of the Green module induced territory at different time points (day: 3,4,5,6,7,8,9,10,11,12,13,14), data are displayed as mean and individual points (n=2 biologically independent samples).

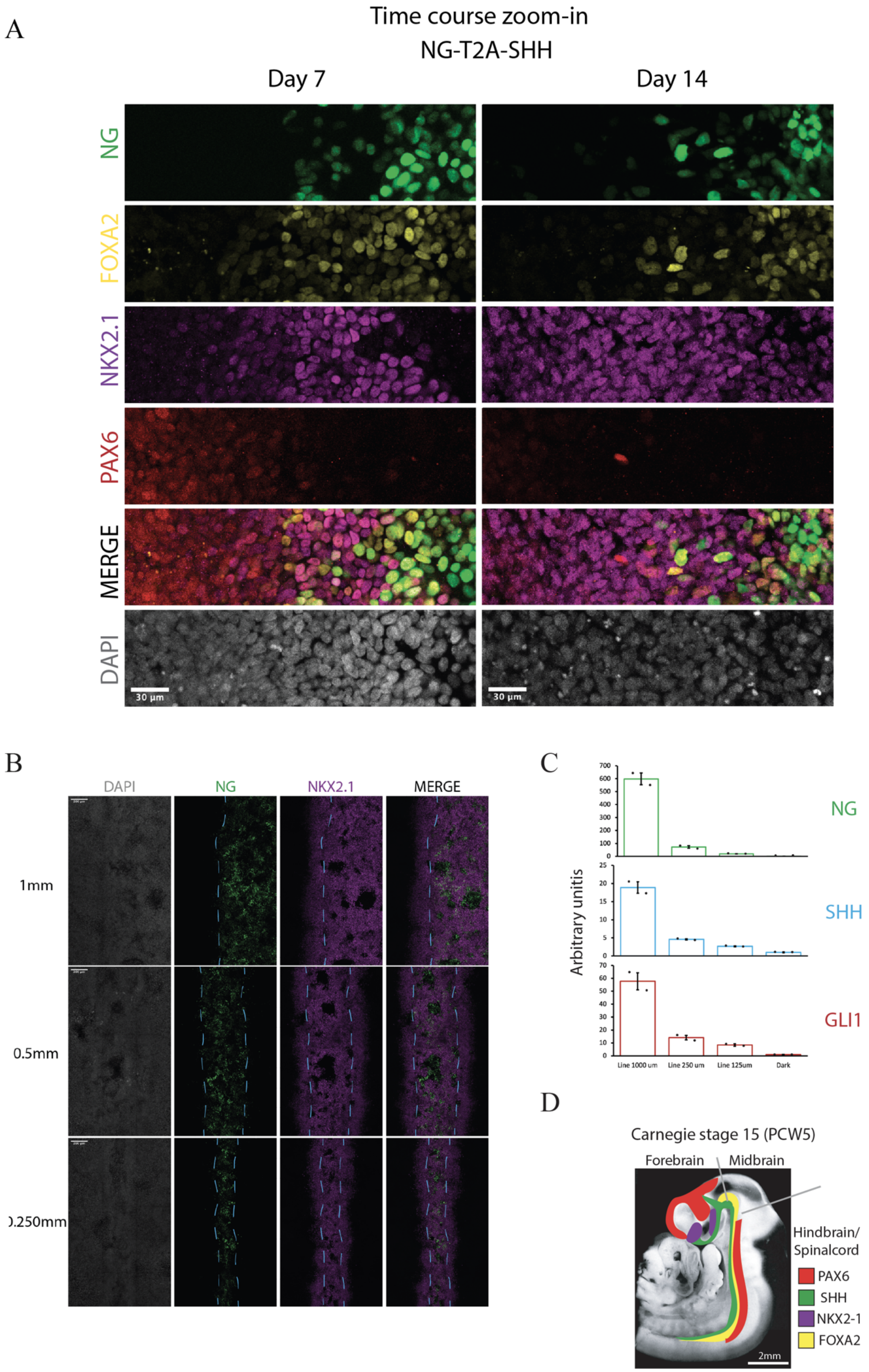
A
Time course zoom-in
NG-T2A-SHH
Day 7
Day 14
NG
FOXA2
NKX2.1
PAX6
MERGE
DAPI
30 µm
30 µm
B
DAPI
NG
NKX2.1
MERGE
1mm
0.5mm
0.250mm
C
NG
SHH
GLI1
Arbitrary unitis
Line 1000 um
Line 250 um
Line 125um
Dark
D
Carnegie stage 15 (PCW5)
Forebrain
Midbrain
Hindbrain/
Spinalcord
PAX6
SHH
NKX2-1
FOXA2
2mm

Suppl. Fig 4: Stimulus-dependent expression of SHH targets upon light induction

(A) Immunostaining at higher magnification showing details of ventral fates induced in proximity of the NG-T2A-SHH organizer at day 7 and 14. DAPI-Grey, NG-Green, FOXA2-Yellow, NKX2-1- Magenta, PAX6-Red and Merge-Composite. Scale bar: 30µm. **(B)** Fluorescence images of large fields of view showing consistent patterning along the light-induced line of SHH at day 14. Color code is: NG-T2A-SHH-Green (Organizer), NKX2-1-Magenta (Ventral) and Merge-Composite. Dashed cyan line indicate the border of SHH producing cells. Scale bar: 200µm. **(C)** qRT-PCR showing the mRNA levels of NG (Green module), SHH, and the SHH-responsive gene GLI1, in samples spatially activated with light using a photo-mask of: 1000µm, 250µm and 125µm. NG, SHH, and its downstream target correlate with the area of induction. Histograms display average, SD and individual points (n=2 biologically independent samples). D) Carnegie collection image featured with a color-coded scheme of the human sagittal section Carnegie stage 15 (PCW5) for specific markers (SHH-Green, NKX2-1-Magenta, PAX6-Red, FOXA2-Yellow), dashed lines delineate Forebrain, Midbrain, and Hindbrain / Spinal cord boundaries. Scale bar = 2mm.

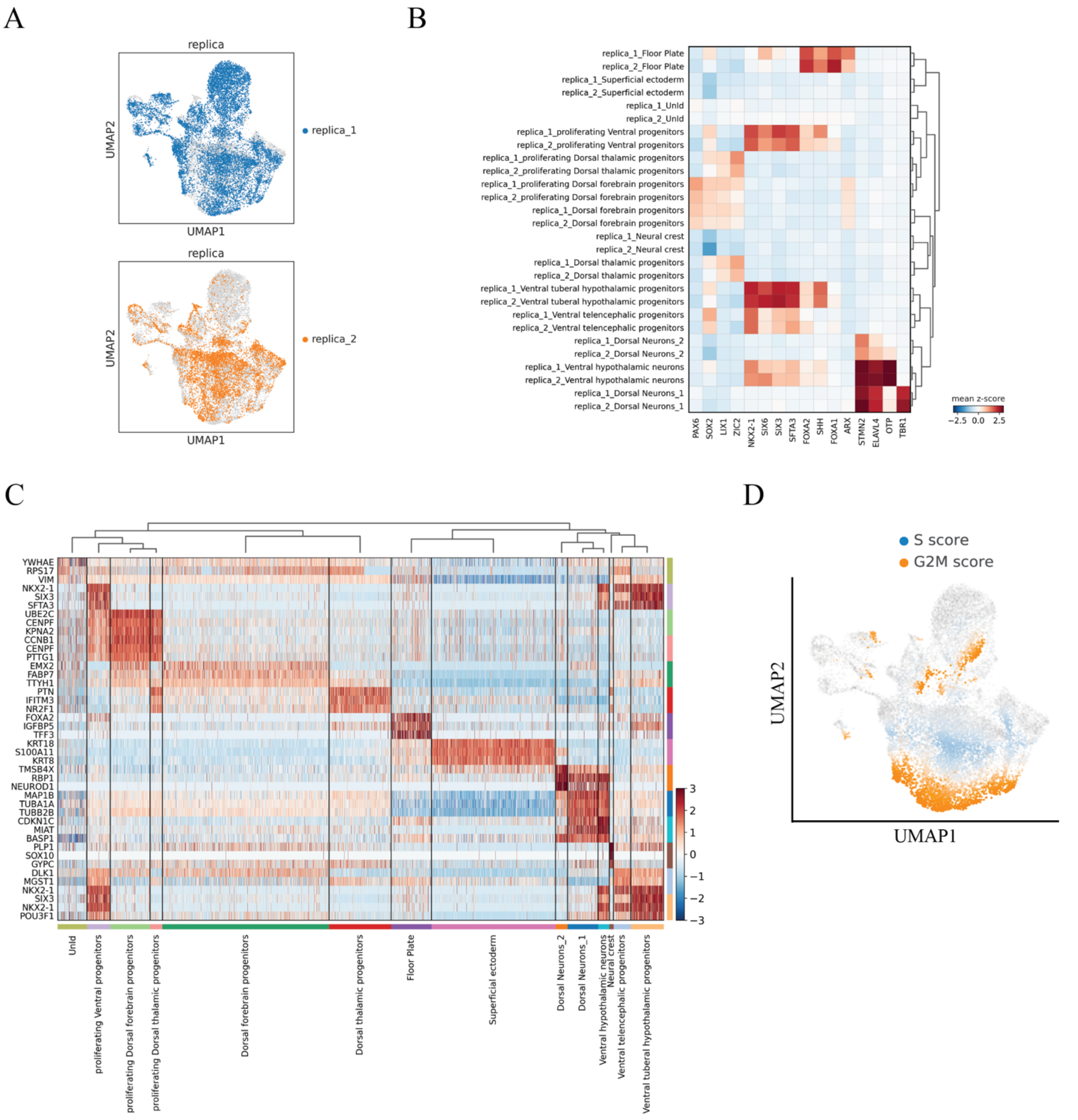

Suppl. Fig 5: scRNA-seq transcriptomic analysis of SHH-induced fates

(A) UMAP plot of the two-biological replica of *in-vitro* light induced SHH cells at day 14. The two replicas integrate in the UMAP space and display the same cellular populations in both replicas. **(B)** z-score heatmap of the two replicas showing the consistent expression in the independent replicas of marker genes used for classification. **(C)** z-score heatmap display the top 5 marker genes for each cell identity in the light induced SHH scRNA-seq dataset. **(D)** scVelo calculated cell cycle prediction were embedded in the UMAP space and used for classification of the proliferating cells.

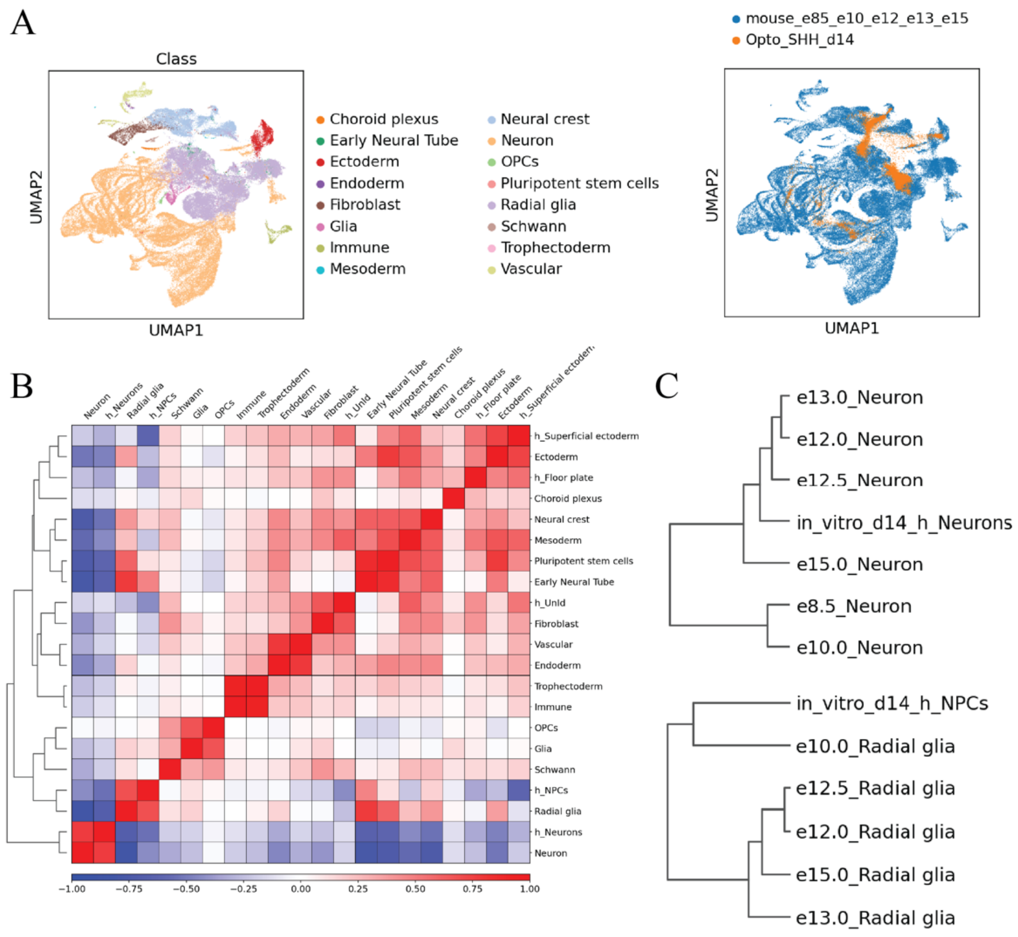

Suppl. Fig 6: Conserved gene expression between light-induced synthetic tissue and their mouse embryonic counterparts

(A) UMAP plot of the mouse brain dataset showing cell populations at different time points (E8.5, E10, E12, E12.5, E13, and E15), UMAP plot shows the mouse dataset annotation “Class” (top left). UMAP plot of the human *in-vitro* light induced SHH cells integrated with the mouse dataset in defined clusters (top right). The human cells mostly integrate with the E10 time points in the Radial glia population, the Ectoderm, the Neural Crest and Neuronal label annotations. (B) Correlation analysis using the high variable genes of the human-mouse integrated dataset displayed as z-score heatmap using the mouse “Class” annotation and the human light induced cells, grouped as neural precursor (NPCs), floor plate, neurons and UnId cells. **(C)** Dendrogram analysis shows clustering of the *in-vitro* derived human cells grouped as NPCs and Neurons, with specific time points of mouse brain development.

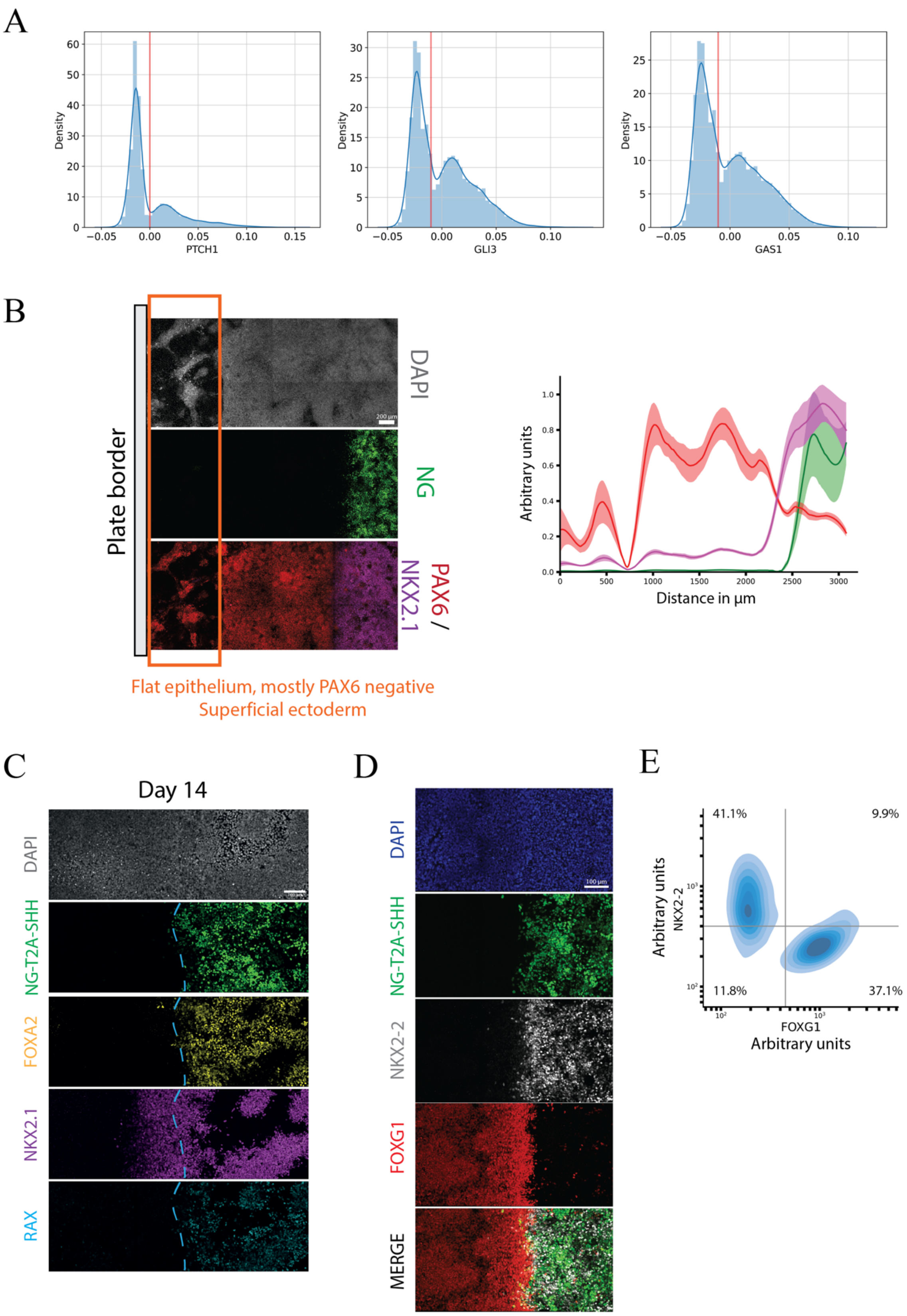
A
Density
PTCH1
GLI3
GAS1
B
Plate border
DAPI
NG
PAD6 /
NKX2.1
Arbitrary units
Distance in µm
Flat epithelium, mostly PAX6 negative
Superficial ectoderm
C
Day 14
DAPI
NG-T2A-SHH
FOXA2
NKX2.1
RAX
D
DAPI
NG-T2A-SHH
NKX2-2
FOXG1
MERGE
E
41.1%
9.9%
11.8%
37.1%
Arbitrary units
NKX2-2
FOXG1
Arbitrary units

Suppl. Fig 7: Identification of SHH responsive cells

(A) Distribution of log normalized scRNA-seq counts for selected genes: (PTCH1, GLI3 and GAS1). Based on the bimodal distribution, specific thresholds are manually selected and displayed as Red vertical bar. Thresholds are used to categorically labels cells as SHH stimulated when GLI3-GAS1^{low}/PTCH1^{high} conditions are valid. **(B)** Large field of view display the well edge that generate most of the PAX-negative cells which correspond to superficial ectoderm and neural crest independently from the light induced SHH organizer. Scale bar = 200 µm (left panel). Cumulative fluorescence intensity analysis (line profile) over the x-axis from the edge of the 96 well. x-axis displays the linear distance in µm. y-axis shows the cumulative fluorescent intensity profile in arbitrary units for each channel. Line profile shows the average (line) and SD (area) for each channel. The line profile is color-coded as the immunofluorescent channels, NG-Green, NKX2-1-Magenta, PAX6-Red at day 14 quantification (n=4 biologically independent samples) (right panel). **(C)** Immunostaining shows the spatial segregation of ventral population arising from a light induced SHH source using an additional hypothalamic marker RAX at day 14. (Green NG, Yellow FOXA2, Magenta NKX2.1, Cyan RAX, Grey DAPI), Scale bar = 100µm. D) Immunostaining shows the spatial segregation of telencephalic (FOXG1) and hypothalamic (NKX2-2) marker in relation to the light induced SHH source of SHH at day 14. (Green NG, Grey NKX2-2, Red FOXG1, Blue DAPI), Scale bar = 100µm. E) Immunostaining single nuclei quantification of NG-T2A-SHH induced cells displayed as density plot at day 14. x-axis report FOXG1 while the y-axis the NKX2-2 fluorescence intensity profile using arbitrary units in a $\log_{10}$ scale.

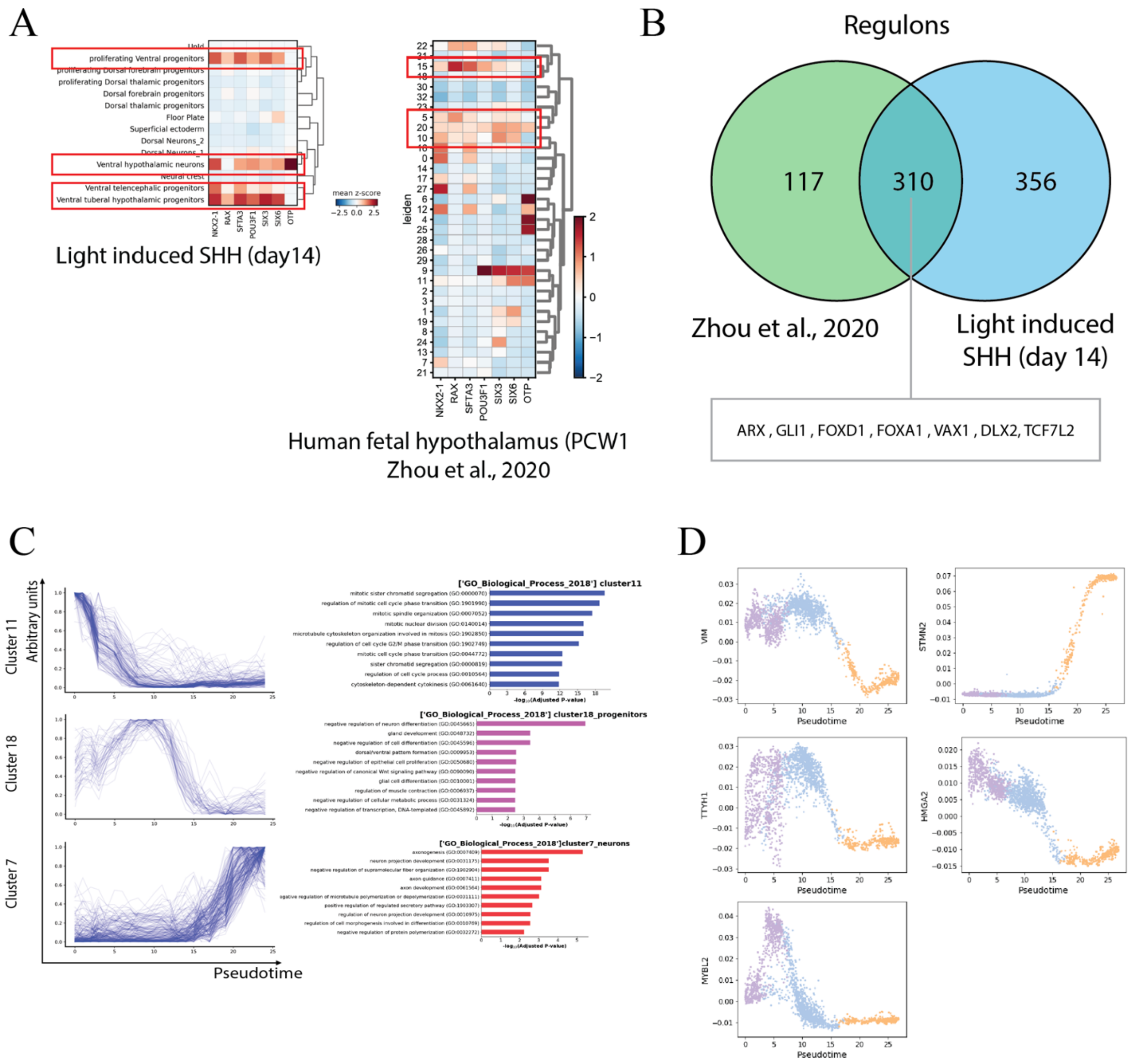

Suppl. Fig 8: Conserved gene expression between light-induced synthetic tissue and their human fetal hypothalamic counterparts

(A) Comparative analysis of hypothalamic markers detected in our light-induced SHH scRNA-seq data, with that of hypothalamic human fetal samples (PCW10). **(B)** Venn-diagram of the active regulons commonly identified using pySCENIC between the fetal hypothalamic dataset and the light induced SHH dataset at day 14. **(C)** Selection of gene patterns identified along the hypothalamic ventral trajectory displayed as line plot over pseudotime, each line represents a gene. The x-axis corresponds to the pseudotime time along the trajectory and the y-axis to the normalized 0-1 scaled gene expression values. Gene patterns have been tested for enrichment of specific categories using GSEAPY-enrichr (GO biological process 2018). **(D)** Pseudotime analysis in the light induced hypothalamic ventral trajectory for selected candidates: VIM, STMN2 are markers

used to validate the progenitor to neuron trajectory (Suppl.Fig 8, top panel); TTYH1,HMGA2 and MYBL2 are candidates described in the fetal hypothalamic development. TTYH1, HMGA2 and MYBL2 are downregulated along the differentiation trajectory in our *in-vitro* model, in agreement to what was recently shown in the human fetal hypothalamus. The scatterplot displays individual cells from the subset of NKX2-1^{+} cells in light induced scRNA-seq dataset. The x-axis reports the pseudotime time along the ventral trajectory and the y-axis the normalized expression values.

List of primers sequence and antibodies:

**Primers sequence:**	***Antibodies list:***
HPRT_FW: GCCCTGGCGTCGTGATTAGT	Anti-PAX6 mouse monoclonal, BD Biosciences 561462, 1:100
HPRT_RV: GGCCTCCCATCTCCTTCATCA	Anti-TTF1 antibody [EP1584Y], Abcam, (ab76013), 1:500
MAGNETS_FW: CTCTGACAGATGCCAGGACA	Anti-HNF-3BETA/FOXA2, Neuromics, GT15186, 1:200
MAGNETS_RV: GATCAGCATTCTCCCACCAT	Anti-NKX2-2, DSHB, 74.5A5, 1:200
CAG_FW: GCTTCGAATTCTGCAGTCGACCG	Anti-FOXG1 [EPR18987], Abcam, ab196868, 1:100
NEON_GREEN_RV: GCTGGGAGAGAGGCCATGTTATCC	Anti-OTP [EPR22178-17], Abcam, ab254267, 1:200
GLI1_FW: TGGCATCCGACAGAGGTGAG	Anti-SIX6, Abcam, ab251658, 1:200
GLI1_RV: CAGTTATGGGCCAGCCAGAGA	Anti-Rx, Takara, M229, 1:200
GLI3_FW: TTGCACAAAGGCCTACTCGAGACT	
GLI3_RV: CTTGTTGCAACCTTCGTGCTCACA	
SHH_FW: AATGTGGCCGAGAAGACCC	
SHH_RV: AGATGGCCAAAGCGTTCAAC	
ATP5O_FW: ACTCGGGTTTGACCTACAGC	
ATP5O_RV: GGTACTGAAGCATCGCACCT	
FOXA2_FW: CCGTTCTCCATCAACAACCT	
FOXA2_RV: GGGGTAGTGCATCACCTGTT	
NKX2-1_FW: AGGGCGGGGCACAGATTGGA	
NKX2-1_RV: GCTGGCAGAGTGTGCCCAGA	
