# Supplementary material for: Self-organization of human dorsal-ventral forebrain structures by light induced SHH
Source: Nat Commun. 2021 Nov 19;12:6768. doi: 10.1038/s41467-021-26881-w (PMC8604999; doi:10.1038/s41467-021-26881-w)
Supplement: Supplementary file 3 — Reporting Summary [file 41467_2021_26881_MOESM3_ESM.pdf]

nature portfolio

Corresponding author(s): Brivanlou A.H.

Last updated by author(s): Oct 1st, 2021

Reporting Summary

Nature Portfolio wishes to improve the reproducibility of the work that we publish. This form provides structure for consistency and transparency in reporting. For further information on Nature Portfolio policies, see our Editorial Policies and the Editorial Policy Checklist.

Statistics

For all statistical analyses, confirm that the following items are present in the figure legend, table legend, main text, or Methods section.

n/a	Confirmed	
☐	☒	The exact sample size (*n*) for each experimental group/condition, given as a discrete number and unit of measurement
☐	☒	A statement on whether measurements were taken from distinct samples or whether the same sample was measured repeatedly
☒	☐	The statistical test(s) used AND whether they are one- or two-sided<br>*Only common tests should be described solely by name; describe more complex techniques in the Methods section.*
☒	☐	A description of all covariates tested
☒	☐	A description of any assumptions or corrections, such as tests of normality and adjustment for multiple comparisons
☐	☒	A full description of the statistical parameters including central tendency (e.g. means) or other basic estimates (e.g. regression coefficient) AND variation (e.g. standard deviation) or associated estimates of uncertainty (e.g. confidence intervals)
☒	☐	For null hypothesis testing, the test statistic (e.g. *F*, *t*, *r*) with confidence intervals, effect sizes, degrees of freedom and *P* value noted<br>*Give P values as exact values whenever suitable.*
☒	☐	For Bayesian analysis, information on the choice of priors and Markov chain Monte Carlo settings
☒	☐	For hierarchical and complex designs, identification of the appropriate level for tests and full reporting of outcomes
☒	☐	Estimates of effect sizes (e.g. Cohen's *d*, Pearson's *r*), indicating how they were calculated

Our web collection on statistics for biologists contains articles on many of the points above.

Software and code

Policy information about availability of computer code

Data collection	All confocal images were acquired by Zeiss Zen (Black edition, 2017) software. Fiji_V2 (Version 2.1.0/1.53c) was used for image display and formatting.
Data analysis	Image analyses and quantifications were performed using czifile (2019.7.2), matplotlib (3.2.2), seaborn (0.11.2), numpy (1.19.5), scikit-image (0.16.2), pandas (1.1.5) libraries in python 3.7. For scRNA-seq, after sequencing, Cell Ranger (v2.0.2) software pipeline was used to align FASTQ files to the hg19 genome. scRNA-seq data was analysed using Scanpy (1.8.1), Kallisto Bustools (0.24.4), pySCenic (0.11.2), scVelo (v0.2.2), scikit-learn (0.22), magic-impute (3.0.0), scprep (1.1.0), gseapy (0.10.5) in python 3.7 as indicated in the Methods section.

For manuscripts utilizing custom algorithms or software that are central to the research but not yet described in published literature, software must be made available to editors and reviewers. We strongly encourage code deposition in a community repository (e.g. GitHub). See the Nature Portfolio guidelines for submitting code & software for further information.

Data

Policy information about availability of data

All manuscripts must include a data availability statement. This statement should provide the following information, where applicable:

- Accession codes, unique identifiers, or web links for publicly available datasets
- A description of any restrictions on data availability
- For clinical datasets or third party data, please ensure that the statement adheres to our policy

All data generated in this study are included in this published article (and its supplementary files information). Accession codes are indicated in the text and scRNA-seq data are deposited on GEO database, under the accession number GSE163505. External dataset used in this study are listed in the "data availability" section.

Field-specific reporting

Please select the one below that is the best fit for your research. If you are not sure, read the appropriate sections before making your selection.

☒ Life sciences ☐ Behavioural & social sciences ☐ Ecological, evolutionary & environmental sciences

For a reference copy of the document with all sections, see nature.com/documents/nr-reporting-summary-flat.pdf

Life sciences study design

All studies must disclose on these points even when the disclosure is negative.

Sample size	Sample size was not a priori determined, imaging data are the result of at least 3 different field of view that have been replicated at least in two independent experiments.
Data exclusions	Imaging of areas with no DAPI staining where excluded from quantification.
Replication	Reported results were repeated and confirmed in at least two independent experiments.
Randomization	No randomization methods were used this work, comparative analysis have been conducted in parallel experiments, for time course experiments images were acquired following chronological order.
Blinding	No blinding methods were used this work. The same investigator set up and analyzed the data.

Reporting for specific materials, systems and methods

We require information from authors about some types of materials, experimental systems and methods used in many studies. Here, indicate whether each material, system or method listed is relevant to your study. If you are not sure if a list item applies to your research, read the appropriate section before selecting a response.

Materials & experimental systems

n/a	Involved in the study	
☐	☒	Antibodies
☐	☒	Eukaryotic cell lines
☒	☐	Palaeontology and archaeology
☒	☐	Animals and other organisms
☒	☐	Human research participants
☒	☐	Clinical data
☒	☐	Dual use research of concern

Methods

n/a	Involved in the study	
☒	☐	ChIP-seq
☒	☐	Flow cytometry
☒	☐	MRI-based neuroimaging

Antibodies

Antibodies used

Anti-PAX6 mouse monoclonal, BD Biosciences 561462, 1:100 Clone O18-1330
Anti-TTF1 antibody [EP1584Y], Abcam, (ab76013), 1:500
Anti-HNF-3BETA/FOXA2, Neuromics, GT15186, 1:200
Anti-NKX2-2, DSHB, 74.5A5, 1:200
Anti-FOXG1 [EPR18987], Abcam, ab196868, 1:100
Anti-OTP [EPR22178-17], Abcam, ab254267, 1:200
Anti-SIX6, Abcam, ab251658, 1:200
Anti-Rx, Takara, M229, 1:200

Validation

Anti-PAX6 mouse monoclonal, BD Biosciences: human reactivity validated by manufacturer also (Chambers et al. (2009) Nat Biotech
Anti-TTF1 antibody [EP1584Y], Abcam, (ab76013): human reactivity validated by manufacturer also (Radonijic et al. (2014) Front Neuroanat
Anti-HNF-3BETA/FOXA2, Neuromics, GT15186: human reactivity validated by manufacturer
Anti-NKX2-2, DSHB, 74.5A5: human reactivity validated by manufacturer also (Briscoe et al. (1997) Cell
Anti-FOXG1 [EPR18987], Abcam, ab196868: human reactivity validated by manufacturer, also Velasco et al., 2019 Nature
Anti-OTP [EPR22178-17], Abcam, ab254267: human reactivity validated by manufacturer
Anti-SIX6, Abcam, ab251658: human reactivity validated by manufacturer
Anti-Rx, Takara, M229: human reactivity validated by manufacturer

Eukaryotic cell lines

Policy information about cell lines

Cell line source(s)	The RUES1 (NIH#0012) and RUES2 (NIH#0013) cell lines were created in our lab and are listed in the NIH Human Embryonic Stem Cell Registry. They were derived under approval from the Tri-Institutional Stem Cell Initiative Embryonic Stem Cell Research Oversight (Tri-SCI ESCRO) Committee, an independent committee charged with oversight of research with human pluripotent stem cells and embryos to ensure conformance with University policies, and guidelines from the U.S. National Academy of Sciences (NAS).
Authentication	Authentication The cell lines were derived in our lab, karyotype (XX or XY genetic identity) was used to distinguish between RUES1 and RUES2.
Mycoplasma contamination	All cell lines tested negative for mycoplasma contamination upon routine luminescence testing every 4 months.
Commonly misidentified lines (See ICLAC register)	No commonly misidentified cell lines were used.
